# Lipid chaperones and associated diseases: a group of chaperonopathies defining a new nosological entity with implications for medical research and practice

**DOI:** 10.1007/s12192-020-01153-6

**Published:** 2020-08-27

**Authors:** Antonella D’Anneo, Celeste Caruso Bavisotto, Antonella Marino Gammazza, Letizia Paladino, Daniela Carlisi, Francesco Cappello, Everly Conway de Macario, Alberto J. L. Macario, Marianna Lauricella

**Affiliations:** 1 Department of Biological, Chemical and Pharmaceutical Sciences and Technologies (STEBICEF), Laboratory of Biochemistry, University of Palermo, 90127 Palermo, Italy; 2 Department of Biomedicine, Neurosciences and Advanced Diagnostics (BIND), Institute of Anatomy, University of Palermo, 90127 Palermo, Italy; 3 Euro-Mediterranean Institute of Science and Technology (IEMEST), 90139 Palermo, Italy; 4 Department of Biomedicine, Neurosciences and Advanced Diagnostics (BIND), Institute of Biochemistry, University of Palermo, 90127 Palermo, Italy; 5 Department of Microbiology and Immunology, School of Medicine, University of Maryland at Baltimore-Institute of Marine and Environmental Technology (IMET), Baltimore, MD 21202 USA

**Keywords:** Fatty acid–binding proteins, Lipid chaperones, Lipid chaperone-associate pathologies, Chaperonopathies, Chaperonotherapy

## Abstract

Fatty acid–binding proteins (FABPs) are lipid chaperones assisting in the trafficking of long-chain fatty acids with functions in various cell compartments, including oxidation, signaling, gene-transcription regulation, and storage. The various known FABP isoforms display distinctive tissue distribution, but some are active in more than one tissue. Quantitative and/or qualitative changes of FABPs are associated with pathological conditions. Increased circulating levels of FABPs are biomarkers of disorders such as obesity, insulin resistance, cardiovascular disease, and cancer. Deregulated expression and malfunction of FABPs can result from genetic alterations or posttranslational modifications and can be pathogenic. We have assembled the disorders with abnormal FABPs as chaperonopathies in a distinct nosological entity. This entity is similar but separate from that encompassing the chaperonopathies pertaining to protein chaperones. In this review, we discuss the role of FABPs in the pathogenesis of metabolic syndrome, cancer, and neurological diseases. We highlight the opportunities for improving diagnosis and treatment that open by encompassing all these pathological conditions within of a coherent nosological group, focusing on abnormal lipid chaperones as biomarkers of disease and etiological-pathogenic factors.

## Introduction

Fatty acid–binding proteins (FABPs) are a class of chaperones that bind and assist lipids in their activities, particularly their migration to different cellular compartments (Furuhashi and Hotamisligil 2008). Interest in these chaperones is rising because their abnormalities have been correlated with human diseases. Here, we discuss the involvement of FABPs in the development and progression of metabolic syndrome, cancer, and neurodegeneration. We propose that diseases in which the lipid chaperones are quantitatively and/or qualitatively abnormal, probably playing an etiopathogenic role in some cases, can be classified in a distinct nosological group similar to, but separate from, that encompassing the chaperonopathies caused by abnormalities in protein chaperones.

## FABPs in health and disease

**Fig. 1:**
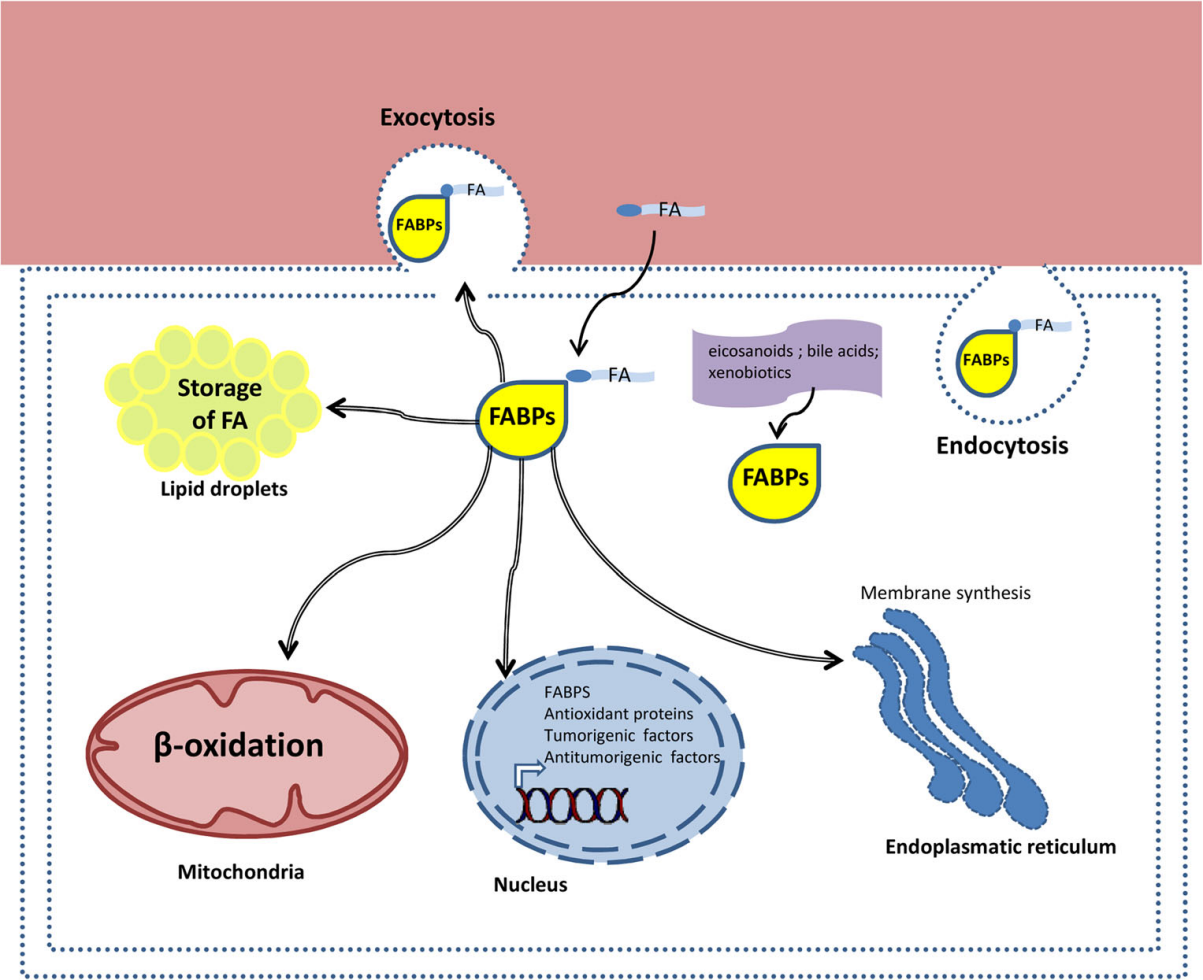
The lipid chaperones FABPs reside and function in various cellular compartments: cytosol, nucleus, mitochondria, endoplasmic reticulum, lipid droplets, and endocytosis and exocytosis vesicles. They also occur extracellularly, for example in the blood. FABPs bind fatty acids and other molecules (e.g., bile acids) and usher them to different intracellular and extracellular compartments. In this way, FABPs can contribute to the (a) β-oxidation of free fatty acids in the mitochondria, (b) regulation of transcription in the nucleus, (c) synthesis of biological membrane components in the endoplasmic reticulum, (d) storage and degradation of triglyceride in lipid droplets, (e) regulation of enzymatic activity in the cytosol, and (e) export of free fatty acids (FA) into the blood. Genetic or acquired abnormalities of lipid chaperones can contribute to the initiation and progression of diseases, the FABP chaperonopathies (see text and Fig. 2). Abbreviations and code: FABP, fatty acid–binding protein; FA, fatty acid; broken double line, plasma-cell membrane; colored area on top, circulating blood

Lipids play multiple roles in various cellular compartments, Fig. 1. They are not only important structural components of the cell membrane but also function as energy source undergoing oxidation in mitochondria and peroxisomes or are stored in the cytoplasm as lipid droplets (Meyers et al. 2017). Lipids act as intracellular and extracellular signaling molecules regulating metabolic functions and inflammation (Jarc and Petan 2020). In the nucleus, lipids can regulate expression of genes related to lipid and carbohydrate metabolism (González-Becerra et al. 2019).

Lipids are hydrophobic and, consequently, they require chaperones, namely proteins that bind lipids, for their trafficking in the aqueous environment of cells, cellular compartments, and extracellular space typical of living organisms. Intracellular lipid-binding proteins (iLBPs) are a group of low molecular mass proteins, including FABP, retinol (CRBP), and retinoic acid (CRABP)-binding proteins, which are involved in the intracellular transport of fatty acids and other hydrophobic molecules (Schaap et al. 2002). In addition, two members of the Hsp70 family of protein chaperones, Hsp70 (HSPA1A) and Hsp73 (HSPA8), have been found to bind non-esterified palmitate and stearate in rat liver and rat brain tissues (Guidon Jr. and Hightower 1986a, b). Each chaperone was able to bind non-covalently four molecules of fatty acid per dimer with the two long-chain saturated fatty acids palmitate and stearate present in a one to one molar ratio.

**Fig. 2:**
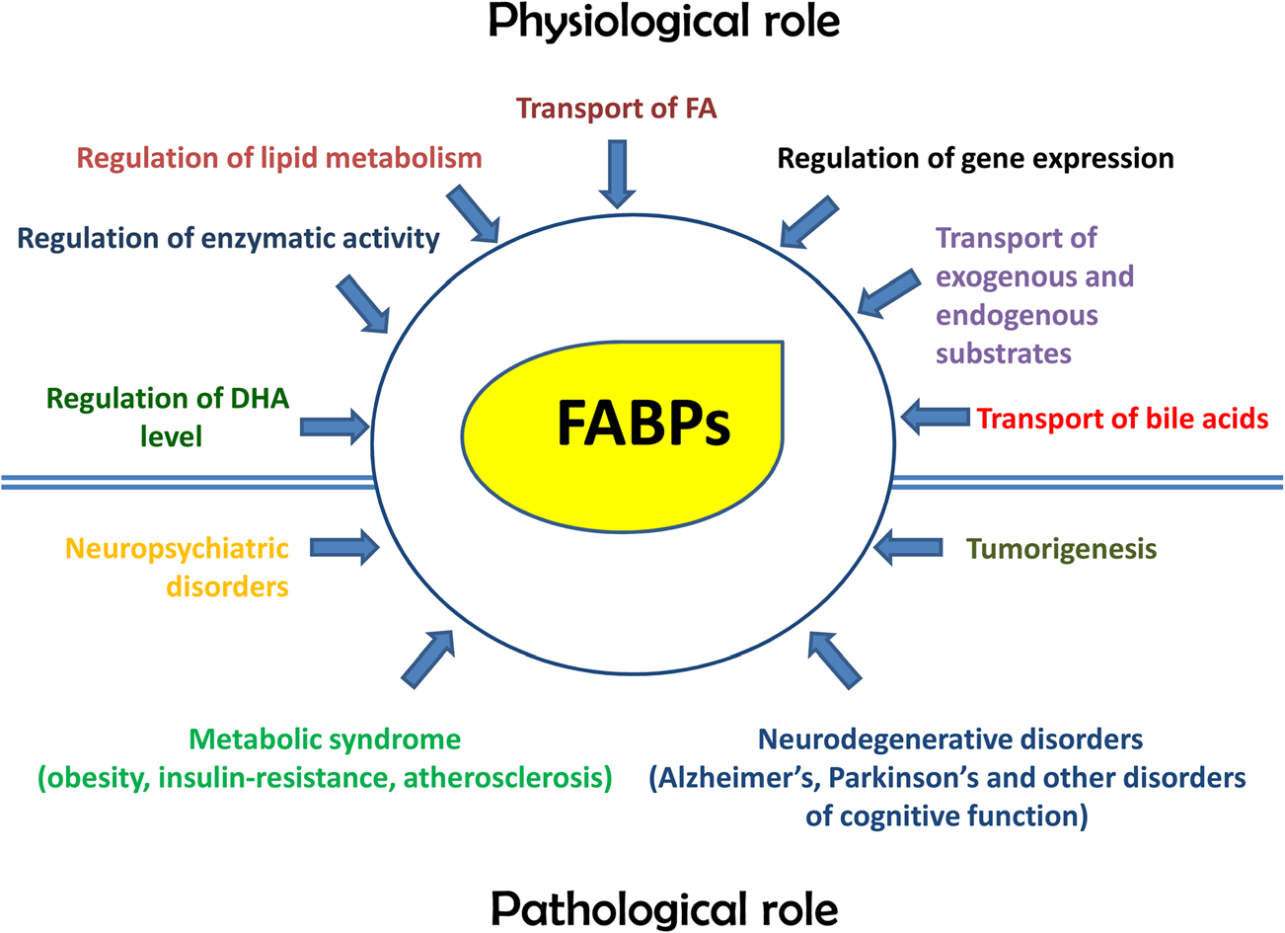
Multiple functions of FABPs and associated chaperonopathies. The functions are physiological when these lipid chaperones are quantitatively and qualitatively normal, or pathogenic when abnormal in one or more of their properties such as structure, function, concentration, intra- or extracellular distribution, and trafficking. The chaperonopathies associated with abnormal lipid chaperones are varied, including metabolic disorders, cancer, and neurological diseases (see text). Abbreviations: FA, fatty acid; DHA, docosahexaenoic acid

FABPs are non-catalytic intracellular binding proteins that associate non-covalently with hydrophobic molecules, including saturated and unsaturated long-chain (C16–C20) fatty acids, eicosanoids, and bile salts and peroxisome proliferators (Hotamisligil and Bernlohr 2015). The first reported FABP was detected in the rat jejunum and described as an intracellular protein of ~ 12 kDa with the ability to non-covalently bind long-chain fatty acids (Ockner et al. 1972). Subsequently, other proteins capable of binding long-chain fatty acids were identified in the liver, myocardium, adipose tissue, and kidney (Ockner and Manning 1974). FABPs function intracellularly like the protein chaperones when these assist the polypeptide maturation and movement in the cytoplasm and organelles. Similarly to protein chaperones, FABPs form non-covalent bonds with their clients, stabilizing lipids in intracellular compartments and favoring their cellular functions. Biochemical studies of FAs binding to FABPs suggested that, upon binding to FABPs, the FAs cross the aqueous cytoplasm in a more favorable energetically state. The main role of FABPs is to mediate the traffic of lipids in the various cellular compartments such as mitochondria, peroxisomes, nucleus, endoplasmic reticulum, and lipid droplets for oxidation, membrane synthesis, signaling, and storage (Furuhashi and Hotamisligil 2008). FABPs escort free fatty acids to meet transcriptional factors like the peroxisome proliferator-activated receptors (PPARs) in the nucleus to regulate gene expression, and bind eicosanoids protecting them from peroxidation (Tan et al. 2002; Furuhashi and Hotamisligil 2008). In short, FABPs play various roles, including binding and ushering fatty acids toward their physiological destinations and assisting in the transcriptional regulation of a range of genes (Figs. 1 and 2).

FABPs are a set of 14–15 kDa proteins, including nine isoforms: liver (L-FABP/FABP1), intestinal (I-FABP/FABP2), heart (H-FABP/FABP3), adipocyte (AFABP/FABP4/aP2), epidermal (E-FABP/FAPB5/mal1), ileal (il-FABP/FABP6), brain (E-FABP/FABP7), myelin (M-FABP/FABP8), and testis (T-FABP/FABP9) (Smathers and Petersen 2011). In addition, another isoform, FABP12, has been reported but its function is still unknown (Liu et al. 2008).

The different isoforms are named for the organ in which they were first identified and are more abundant, but their expression profiles are not exclusive for a specific organ. For example, FABP1 is expressed not only in the liver but also in the intestine, pancreas, lung, and stomach (He et al. 2010). Table 1 summarizes the main functions of the FABPs and the diseases associated with their dysfunction. Lipid chaperones are implicated in a range of diseases probably as part of the pathogenic mechanisms or at least as biomarkers, Fig. 2. If a FABP participates in the pathogenic mechanism, the condition may be classified as a true chaperonopathy, as it is done for diseases in which molecular chaperones for proteins are etio-pathogenic factors (Macario and Conway de Macario 2005). FABP chaperonopathies can, thus, be considered a distinct nosological group encompassing all pathological conditions in which defective, malfunctioned lipid chaperones contribute to the mechanism of disease.

**Table 1:** Fatty acid–binding proteins (FABPs): characteristics and associated pathologies

Name	Localization	Role	Pathology	aa (Chr)	Reference
*FABP1*; L-FABP; FABPL	Liver, alveolar epithelium cells small intestine, colon, duodenum, kidney	Bind FA, bile acids and exogenous substrates	↑Liver damage (serum) ↑Gastric cancer ↓Hepatocarcinoma	127aa (Chr. 2)	Smathers and Petersen 2011; Karvellas et al. 2017; Santos and Schulze 2012
*FABP2*; FABPI; I-FABP	Small intestine, duodenum, colon	Transport of exogenous FA; modulation of cell growth and proliferation	↑Colon cancer ↑Acute pancreatitis	132 aa (Chr. 4)	Hu et al. 2013; Goswami et al. 2017
*FABP3*; MDGI; FABP11; H-FABP; M-FABP; O-FABP	Heart, neurons, glia, kidney, prostate	Transport of FA and other lipophilic substrates; modulation of cell growth and proliferation	↑Heart failure (serum) ↑Uveal, gastric, and brain tumor ↑NSCLC ↓Breast cancer ↑Neurodegenerative disorders	133 aa (Chr. 1)	Zimmerman and Veerkamp 1998; Song et al. 2012; Tang et al. 2016; Matsumata et al. 2016; Chiasserini et al. 2017; Kawahata et al. 2019
*FABP4*; aP2; ALBP; AFABP; A-FABP; HEL-S-104	Fat, liver, limb, whole brain, placenta	Transport of FA in intracellular compartment; Export of FA in plasma; Regulation of lipid metabolism	↑Obesity and metabolic syndrome (plasma) ↑Cardiovascular disease ↑Atherosclerosis ↑Mammary tumor risk ↓Prostate and liver cancer	132 aa (Chr. 8)	Amri et al. 1991; Makowski et al. 2001; Boss et al. 2015; Prentice et al. 2019; Shen et al. 1999; Chan et al. 2010; Tang et al. 2016; Hammamieh et al. 2005; Hancke et al. 2010; Boiteux et al. 2009; Zhong et al. 2018
*FAPB5*; EFABP; KFABP; E-FABP; PAFABP; PA-FABP	Esophagus, fat, colon, skin, colon, lung, limp node, neurons, and glia	Transport of FA; regulation of DHA level in the brain neuron development	↑Breast and prostate cancers ↑Psoriasis ↓Cognitive deterioration	135 aa (Chr. 3)	Adamson et al. 2003; Matsumata et al. 2016; Pan et al. 2018
*FAPB6*; ILBP; I-15P; I-BAP; ILBP3; ILLBP; I-BABP; I-BALB	Small intestine	Transport of FA; correct absorption and transport of bile acids	↑Colon cancer ↑Renal-cell carcinoma	128aa (Chr. 5)	Zhang et al. 2019; Nagao et al. 2018
*FABP7*; MRG; BLBP; FABPB; B-FABP	Brain, neurons, glia, skin, salivary gland, fat	Transport of FA; plays a fundamental role in the formation of radial fiber in the developing brain	↑Tumorigenesis (breast cancer, melanoma, renal carcinoma, cystic carcinoma, and invasive glioma) ↓Neurodegenerative and neuropsychiatric disorders ↑Protective response to BBB disruption	132 aa (Chr. 6)	Liu et al. 2010; Nagao et al. 2018; Matsumata et al. 2016; Pelsers and Glatz 2005; Teunissen et al. 2011; Ebrahimi et al. 2016; Rui et al. 2019
*FABP8*; PMP2; P2; MP2; CMT1G; M-FABP	Brain (myelin sheaths of the peripheral nervous system)	Transport of FA; stabilizes the myelin sheath	↓Dominant demyelinating Charcot-Marie-Tooth neuropathy	132 aa (Chr. 8)	Hong et al. 2016
*FABP9*; PERF; TLBP; PERF15; T-FABP	Testis, spleen, fat brain, endometrium	Transport of FA, fertility (?)	↑Progression and development of prostate cancer.	132aa (Chr. 8)	Al Fayi et al. 2016
*FABP12*	Testis, esophagus, lung, duodenum	germinal lipid metabolism (?)	Not reported	140 aa (Chr. 8)	Liu et al. 2008

Abbreviations: aa, number of amino acids; (Chr.), chromosome number in which the pertinent gene is located; FA, fatty acid(s); NSCLC, non-small cell lung cancer. Symbols: ↑, elevated; ↓, decreased

FABP isoforms share from 15 to 70% sequence identity displaying the same three-dimensional structure with ten antiparallel β strands which are arranged into two orthogonal five-stranded beta-sheets. The β-sheets are capped by two *α*-helices that enclose an internal water-filled cavity, which forms the ligand-binding pocket. FABPs act as chaperones facilitating the intracellular solubilization and transport of FAs via noncovalent interactions. FABPs bind to single long-chain fatty acids except for FABP1, which binds two fatty acids. The isoforms show different ligand specificity, even though they tend to bind long-chain unsaturated fatty acids more strongly than short-chain saturated fatty acids (Storch and Thumser 2010). Hydrophobic molecules bind to FABPs within the β-sheets in the central internal water-filled cavity which functions as ligand-binding pocket (Zimmerman and Veerkamp 2002). This cavity contains the side chains of both hydrophobic and polar amino acids, which can be different in FABP isoforms. The different amino acids in FABP members probably determine the volume of the cavity and the binding specificity. Hydrophobic amino acid residues play a role in ligand affinity and/or specificity by forming Van der Waals bonds with the acyl chain of the bound FA. In FABPs, internal water molecules within the cavity contribute to the displacement of FA and maintain the electrostatic interactions inside the binding cavity (Zimmerman and Veerkamp 2002). X-ray crystallography and nuclear magnetic resonance (NMR) studies showed that FA binds to FABP-binding pocket with the carboxylate group orientated facing inwards. In particular, the carboxylate group of the FA interacts within the water filled cavity with tyrosine and arginine residues (Smathers and Petersen 2011). The two α-helices of FABPs act as a regulatory portal region regulating the entry and release of ligand from the binding pocket (Smathers and Petersen 2011). A conformational change in the portal region occurs during FA binding or release. Interactions of FABP with membranes or other proteins may catalyze this conformational change. It has been shown that the α-helical domain is involved in the regulation of FAs transfer from FABP to membranes (Corsico et al. 1998). This regulation is mediated by collisional interactions of FABPs with membranes and is enhanced by electrostatic interactions between charged lysine residues of the α-helical domain of FABPs and negative charges of phospholipids (Herr et al. 1996; Liou et al. 2002; Liou and Storch 2001). FABPs are also capable of binding other proteins thus regulating their functions. It has been shown that FABP4 can translocate to the nucleus and interact with, and potentially deliver ligands to, PPARγ (Tan et al. 2002), although the functional consequences of this interaction are unknown. Moreover, A-FABP and E-FABP bind hormone-sensitive lipase (HSL) in a fatty acid-dependent manner and this interaction stimulates the activity of the lipolytic enzyme (Jenkins-Kruchten et al. 2003). Thus, FABPs function intracellularly like the protein chaperones when these assist polypeptide maturation and movement in the cytoplasm and organelles. Since FABPs lack a secretory signal sequence, circulating levels of FABPs have been associated to tissue injury (Hotamisligil and Bernlohr 2015). For example, an increase of circulating FABP1 has been correlated with liver damage (Karvellas et al. 2017), and a serum-FABP3 increase has been considered a marker of heart failure with myocardial injury (Rezar et al. 2020). FABP4 is released by adipocytes under fasting conditions, and serum FABP4 acts as an adipokine mediating biological effects in different cell types (Cao et al. 2006). High levels of serum FABP4 have been correlated with obesity and insulin resistance (Simón et al. 2009; Terra et al. 2011; Xu et al. 2006). In addition, deregulated expression of different FABP isoforms has been correlated with cancers and neurological diseases, as discussed below. Abnormal conditions associated with FABPs quantitative and/or qualitative abnormalities will be discussed as chaperonopathies to direct the attention of scientists and medical practitioners toward lipid chaperones as the possible etio-pathogenic factors, and as biomarkers potentially useful for diagnosis and for assessing prognosis and response to treatment. This approach will also unveil clues for developing novel therapeutic means using lipid chaperones as targets or agents.

## Molecular chaperones and chaperonopathies

The protein chaperoning (chaperone) system of an organism consists of the entire set of molecular chaperones, chaperone co-factors, co-chaperones, and chaperone interactors and receptors (Macario and Conway de Macario 2019, 2020). Molecular chaperones are proteins that span a wide range of sizes from the smaller ones with 35 kDa or less to the largest ones of 100–200 kDa and more; many are named heat shock protein (Hsp) and include several families of phylogenetically related members such as for example the small Hsp with the alpha crystallin domain, Hsp40(DnaJ), Hsp70(DnaK), and Hsp90. Specific guidelines for classifying and naming all these chaperones have been proposed, which have contributed to organize information within this complex field (Kampinga et al. 2009).

Although chaperones are typically cytoprotective, they can also be pathogenic if abnormal in structure, function, quantity, or location. Diseases in which a member of the chaperoning system plays an etiological-pathogenic role are the chaperonopathies, which can be genetic or acquired (Macario and Conway de Macario 2005; Marino Gammazza et al. 2014; Rappa et al. 2016). While the former chaperonopathies are associated with genetic variants (e.g., mutation) of chaperone genes, in the acquired chaperonopathies, chaperone genes are normal, but the chaperone protein is not as a consequence of a posttranscriptional event, for instance an aberrant posttranslational modification. Based on the pathogenic mechanism, chaperonopathies can be classified into by excess, by defect, or by mistake (Macario and Conway de Macario 2007a; Macario and Conway de Macario 2007b). Since the chaperoning system is widespread in the body, chaperonopathies are usually systemic diseases that affect numerous tissues and organs and are, therefore, of interest to a wide range of medical specialties. Examples of genetic chaperonopathies are the hereditary motor neuropathy type II and axonal Charcot–Marie–Tooth disease. Their pathogenesis is associated with the mutations K141E and K141N in the Hsp22 (HSPB8) gene, which compromises the structure and function of the chaperone, allowing the formation of aggresomes with pathological protein precipitates that impair proper transport along the axons of the motor neurons (Sun et al. 2010). Other genetic chaperonopathies affect the chaperonins of group I (e.g., Hsp60 (HSPD1)) and II (e.g., CCT (chaperonin-containing TCP-1 polypeptide)). Examples of the former are the hereditary spastic paraplegia 13 (SPG13) and the MitCHAP-60 disease (Pelizaeus-Merzbacher-like), in which the Hsp60 functions are impaired (Bross and Fernandez-Guerra 2016), and a distal sensory neuropathy associated with a mutation in the subunit 5 of CCT (Bouhouche et al. 2006a, b). Annotated lists of genetic and acquired chaperonopathies are included in various publications (Macario et al. 2013; Kakkar et al. 2014; Macario and Conway de Macario 2019, 2020) and in the dedicated continuously updated Web site The Chaperonopathies (http://www.chaperones-pathology.org).

While the preceding considerations apply to the chaperoning system whose components are proteins with polypeptides-proteins as substrates, in this article, we propose to classify the diseases associated with the chaperones that are proteins but with lipids as substrates within a distinct nosological group, the FABP chaperonopathies.

This will facilitate the learning and teaching of these diseases in a coherent fashion, allowing for cross-fertilization of knowledge, conceptual and practical associations, and developing of novel concepts with extensive impact on a variety of conditions seemingly unrelated. This, in turn, should speed up the invention of new diagnostic and therapeutic means applicable to diseases pertinent to diverse areas of medicine.

## FABP chaperonopathies

### Metabolic syndrome

Metabolic syndrome (MS) designates a group of conditions occurring together that increase the risk of developing type 2 diabetes and cardiovascular diseases (Samson and Garber 2014). These conditions include obesity, hyperglycemia, high levels of triglycerides and low high-density lipoprotein (HDL), hypertension, and insulin resistance. The pathogenesis of MS is complex, with obesity and insulin resistance being important contributing factors.

Adipose tissue is an endocrine tissue that releases a variety of hormones called adipokines, which play a critical role in energy homeostasis (Chung and Choi 2020). In adipose tissue of obese individuals, several adipokines are altered (Landecho et al. 2019). Pro-inflammatory factors, such as leptin, tumor necrosis factor α (TNF-α), monocyte chemoattractant protein 1 (MCP-1), resistin, and FABP4, are elevated, while anti-inflammatory factors, e.g., adiponectin, are low, in chronic systemic inflammation, promoting insulin resistance and atherosclerosis (Chung and Choi 2020).

#### FABP4

This chaperone is also known as adipocyte fatty acid–binding protein (AFABP) or adipocyte protein 2 (aP2), and it is highly expressed in adipocytes and macrophages (Amri et al. 1991; Boss et al. 2015; Makowski et al. 2001), Table 1. The level of FABP4 is greatly increased during adipocyte differentiation under the control of different stimuli, including insulin, dexamethasone, and PPARγ agonists (Amri et al. 1991). FABP4 is involved in the transport of fatty acids in intracellular compartments, like mitochondria, endoplasmic reticulum, and nucleus, maintaining adipocyte homeostasis as its activation is linked to nutritional status (Prentice et al. 2019). Adipocytes of FABP4-deficient mice have a decreased lipolysis efficiency, suggesting the chaperone regulates lipolysis in these cells (Shen et al. 1999). Under nutrient deprivation, triglycerides accumulated in lipid droplets are hydrolyzed into glycerol and fatty acids via lipolysis by the sequential activation of adipose triglyceride lipase (ATGL), hormone-sensitive lipase (HLS), and monoglyceride lipase (MGL). FABP4 regulates the lipolytic process via two separate mechanisms: under conditions of lipolysis, FABP4 binds free fatty acids released from triglycerides, favoring their efflux from lipid droplets to the membrane; and in the presence of fatty acids, FABP4 binds and activates, both HSL and comparative geneidentification-58 (CGI-58), the activator of ATGL (Hofer et al. 2015; Shen et al. 1999).

FABP4 is secreted by adipocytes in response to fasting signals (Cao et al. 2013; Mita et al. 2015), while in the feeding state, FABP4 secretion is suppressed by insulin (Prentice et al. 2019). The chaperone is secreted in association with lipolysis by a non-classical secretory pathway. Under lipolysis stimulation, FABP4 is included in the endosomal/lysosomal compartment followed by lysosome exocytosis and release of FABP4 in the extracellular space (Ertunc et al. 2015), Fig. 1. Plasma levels of FABP4 are markedly increased in obese subjects compared to normal weight controls (Xu et al. 2006) and circulating FABP4 levels positively correlate with increased waist circumference, dyslipidemia, and insulin resistance (Choi et al. 2009). Adipose tissue of obese patients undergoes uncontrolled lipolysis as a consequence of insulin resistance (Xu et al. 2006), which might explain the high plasma levels of FABP4 observed in obese patients. Obesity is characterized by a chronic low-grade inflammation (Engin 2017) and adipocytes of obese individuals release chemokines that induce the recruitment of pro-inflammatory macrophages (Wensveen et al. 2015). In addition, pro-inflammatory signals increase the expression of FABP4 in macrophages (Kazemi et al. 2005; Makowski et al. 2005). Since also macrophages release FABP4, although at lower levels than adipocytes, these cells may contribute to increase the serum level of FABP4 in obesity.

PPARγ is a crucial regulator of adipogenesis and insulin responsiveness in adipocytes (Mota de Sá et al. 2015; Huang et al. 2018), and inhibits NF-κB activation, and NF-κB-regulated inflammatory pathways (Makowski et al. 2005). PPARγ increases FABP4 expression in adipocytes and macrophages that is paralleled by PPARγ ubiquitination and proteasomal degradation, resulting in a reduction of the chaperone levels (Siersbaek et al. 2010). Deletion of FABP4 increases PPARγ expression, and lipogenesis and insulin sensitivity in adipocytes (Garin-Shkolnik et al. 2014), suggesting that FABP4-dependent inhibition of PPARγ might favor insulin resistance and induction of inflammatory pathways associated with obesity.

Circulating FABP4 could act as an adipokine regulating various biological processes (Prentice et al. 2019). Serum FABP4 induces insulin secretion in pancreatic β cells (Galic et al. 2010; Wu et al. 2014) and enhances hepatic glucose production via gluconeogenesis (Cao et al. 2013). Moreover, FABP4 decreases the uptake and utilization of glucose in muscles and liver (Maeda et al. 2005). Thus, the elevated levels of circulating FABP4 in obese individuals could induce hyperglycemia and hyperinsulinemia promoting insulin resistance and the development of type 2 diabetes.

Mice deficient in FABP4 are resistant against metabolic abnormalities associated with obesity, including diabetes and atherosclerosis (Boord et al. 2004; Cao et al. 2006). In obese individuals, higher serum FABP4 concentrations correlate with insulin resistance, type 2 diabetes, and endothelial dysfunction (Cabré et al. 2008; Nakamura et al. 2017; Tso et al. 2007). This correlation is supported also by the finding that a monoclonal antibody that targets serum FABP4 lowered fasting blood glucose, increased insulin sensitivity, and reduced fat mass in mouse models (Burak et al. 2015).Thus, high levels of circulating FABP4 are not only to be considered a biomarker of obesity but also the manifestation of a FABP4 chaperonopathy contributing to the causation of the metabolic syndrome.

The levels of FABP4 increase in parallel with differentiation from monocytes to macrophages (Pelton et al. 1999). Various pro-inflammatory stimuli, including lipopolysaccharide, toll-like receptors agonists and oxidized LDL, increase FABP4 levels in macrophages (Kazemi et al. 2005). Mice lacking FABP4 (FABP4^−/−^) are partially protected against the development of atherosclerosis also in obese conditions (Pelton et al. 1999), suggesting that atherosclerosis may be favored by circulating chaperone, perhaps when surpassing certain levels.FABP4 was found in human atherosclerotic plaques and its presence has been associated with vulnerable plaques (Peeters et al. 2011). In coronary plaques FABP4 is expressed in macrophages and increases in these cells the accumulation of cholesterol and the formation of foam cells via inhibition of the ATP-binding cassette A1 (ABCA1) pathway (Makowski et al. 2005). Not only macrophages but also adipocytes of pericardial/perivascular adipose tissue produce FABP4, thus increasing the local level of FABP4 in heart (Furuhashi et al. 2016). This has been correlated with the development of coronary atherosclerosis by promoting vascular inflammation as well as the proliferation and migration of smooth muscle cells (Furuhashi et al. 2016; Fuseya et al. 2017). FABP4 induces in macrophages inflammatory responses through the activation of NF-kB and c-Jun N terminal kinase (JNK) (Hui et al. 2010). Furthermore, FABP4 promotes endothelial dysfunction by decreasing the activation of nitric oxide synthase in vascular endothelial cells (Furuhashi et al. 2016). All these data support the notion that, under certain conditions, FABP4 contributes to pathogenesis of disorders that qualify as chaperonopathies depending on quantitative variations of the chaperone. Future studies should investigate if qualitative changes in the FABP4 molecule are also part of the problem.

Although circulating FABP4 can stimulate intracellular signaling in macrophages, and in endothelial and liver cells, the mechanisms remain unknown. Receptors for FABP4 have not been identified yet, but it has been reported that FABP4 can interact with cytokeratin 1 in the endothelial cell membrane and that this interaction promotes FABP4 cellular uptake and oxidative stress (Saavedra et al. 2015).

FABP4 has higher affinity for polyunsaturated fatty acid (PUFA), such as linoleic acid and linolenic acid, than for saturated ones, suggesting a role of FABP4 as PUFA transporter (Furuhashi et al. 2016). Under oxidative stress, FABP4 undergoes a conformational change that increases its affinity for palmitic acid, a saturated fatty acid (Furuhashi et al. 2016) and obesity is accompanied by oxidative stress in adipose tissue: thus, under these conditions, FABP4 secreted by adipocytes might be modified and increase its affinity for palmitic acid, whose circulating levels increase in high-fat feeding. This would be an example of FABP4 chaperonopathy with a qualitative alteration of the chaperone leading to pathogenesis. The change in FABP4 affinity for palmitic acid induced by oxidative stress could activate inflammatory responses since palmitic acid can stimulate Toll-like receptor 4 (TLR4) signaling (Korbecki and Bajdak-Rusinek 2019). Treatment with recombinant FABP4 in the presence of palmitic acid, but not in its absence, stimulates the activation of inflammatory pathways in macrophages, and endothelial and vascular smooth-muscle cells (Furuhashi et al. 2016). Since all these events are triggered by stimulation of TLR-4 by palmitic acid-bound FABP4, the latter is clearly implicated in pathogenesis.

Posttranslational modification (PTM), including phosphorylation, carbonylation, and acetylation, can occur in FABP4 (Hellberg et al. 2010; Hresko et al. 1990; Xu et al. 2006). However, their significance is still unknown. In addition to the changes in the FABP4 affinity induced by oxidative stress, genetic variants of the FABP4 in humans may be important determinants for cardiometabolic risk, particularly among obese individuals. Alleles that reduce FABP4 expression are protective, while those associated with higher levels of FABP4 increase susceptibility to cardiovascular disease and type 2 diabetes (Chan et al. 2010; Khalyfa et al. 2010; van der Laan et al. 2018). These latter variants would constitute genetic chaperonopathies affecting the FABP4 gene. Obese individuals carrying a T-87C polymorphism in the FABP4 gene, which reduces transcription of FABP4, have decreased dyslipidemia and atherosclerosis (Chan et al. 2010). In addition, the variant rs12539895 of FABP4 has been associated with a reduction of fat in carotid plaques (van der Laan et al. 2018). On the other hand, a sr1054135 polymorphism associated with high plasma levels of FABP4 in both obese and non-obese patients correlates with increased risk of insulin resistance and systemic inflammation (Khalyfa et al. 2010). Similar examples of correlation of polymorphism in the genes encoding the chaperones of proteins with chaperonopathies clinically manifest exist (Macario et al. 2013). The same concept is therefore applicable to FABPs, as chaperonopathies. Moreover, the treatment of chaperonopathies includes positive and negative chaperonotherapy (Macario and Conway de Macario 2007a; Cappello et al. 2014). The former applies to chaperonopathies by defect, namely when there is a quantitative or qualitative deficiency of a chaperone, whereas negative chaperonotherapy pertains to cases in which a chaperone is pathogenic and must be blocked or eliminated (Macario and Conway de Macario 2007b). An example of this is the inhibition of the chaperone FABP4 with the inhibitor BM5309403. This is pertinent to the management of the metabolic syndrome in which FABP4 plays a causative role. BM5309403 is a specific FABP4 inhibitor that interacts with the fatty acid–binding pocket of FABP4 and prevents the binding of endogenous fatty acids (Furuhashi et al. 2007), improving glucose metabolism and insulin sensitivity in diabetic mouse models (Burak et al. 2015). Moreover, BM5309403 reduces accumulation of cholesterol and foam cell formation in macrophages (Furuhashi et al. 2007; Miao et al. 2015).

Briefly, several studies have revealed a correlation between FABP4 quantitative or qualitative changes, genetic or acquired, and metabolic abnormalities leading to disease. This correlation has been revealed not only for FABP4 and metabolic disorders, but also for other FABPs and diseases, as discussed in the following sections.

### Cancer

Some FABPs that are increased extracellularly and in circulation are implicated in tumor progression. Their profile of expression has been found deregulated during early tumor transformation and expansion (Amiri et al. 2018).

#### FABP1

This chaperone expressed mainly in hepatocytes, and in intestinal and alveolar epithelium cells, can bind a range of molecules beyond fatty acids (FAs), for example xenobiotics, benzodiazepines, β-blockers, and non-steroidal anti-inflammatory drugs (NSAIDs), Table 1. FABP1 is elevated in gastric cancer accompanied with high levels of fatty acid synthase (FAS), an enzyme involved in fatty acid synthesis to support energetic requirements of tumor growth, survival, invasion, and angiogenesis (Santos and Schulze 2012). This would be an example of quantitative FABP1 chaperonopathy, or of chaperonopathy by mistake, consisting of a normal chaperone FABP1 in this case, being “mistakenly” involved in a pathway favoring carcinogenesis (Macario and Conway de Macario 2007b). This kind of chaperonopathies are candidates for treatment with negative chaperonotherapy, in which the “mistaken” chaperone is inhibited or eliminated.

In contrast, bad prognosis of hepatocellular adenoma was associated with a decrease of FABP1 and increase of hepatocyte nuclear factor 1 α (HNF-1α) and low levels of FABP1 were also found in hepatocarcinoma (HCC) patients (Amiri et al. 2018).

#### FABP2

This chaperone is involved in the absorption and transport of dietary long chain fatty acids and is implicated in intestinal tumorigenesis, Table 1. The FABP2 variant Ala54Thr FABP2 increases the risk of colon cancer development in patients with pronounced hyperinsulinemia and obesity, but, for this to happen, coincidence with other risk factors seems necessary (Hu et al. 2013). Although FABP2 chaperonopathy is not firmly established as a carcinogenic factor in patients with colon cancer and hyperinsulinemia and obesity, this issue deserves further investigation, considering the possibility of applying chaperonotherapy to treat these very sick patients.

#### FABP3

There is conflicting evidence about the role of FABP3 in carcinogenesis, Table 1. Some data support the role of FABP3 as a tumor suppressor capable of counteracting breast cancer progression (Zimmerman and Veerkamp 1998) and growth of embryonic cancer cells (Song et al. 2012). However, high levels of FABP3 were found in metastasis of uveal tumors and its presence was associated with increased aggressiveness of some gastric cancers (Hashimoto et al. 2004). High expression of FABP3, at both mRNA and protein levels, in conjunction with FABP4, indicate poor prognosis in patients with non-small cell lung cancer (NSCLC), suggesting an oncogenic role of this chaperone (Tang et al. 2016).

#### FABP4

Quantitative changes of this chaperone are characteristic of white-to-brown adipocyte differentiation (white-to-brown conversion), and its expression is abundant during the monocyte-to-activated macrophage transition (Amri et al. 1991; Boss et al. 2015; Makowski et al. 2001), Table 1. FABP4 is produced and released into the blood by adipocytes, a phenomenon correlated with obesity (Xu et al. 2006), which indicates that this chaperone can be pathogenic in the metabolic syndromes, as discussed earlier. In addition, FABP4 can also play a role in carcinogenesis, as suggested by data that demonstrate a correlation between levels of the chaperone and tumor progression (Hammamieh et al. 2005; Tang et al. 2016). In obese breast-cancer patients, in which adipokines such as leptin and resistin known to be related to obesity and that increase mammary tumor risk, an elevation of FABP4 correlated with tumor size and stage, and with lymph node invasion was found (Hancke et al. 2010).

For prostatic cancer, contradicting evidence has been described about the role of FABP4. A very low content of FABP4 was found in human prostate cancer cells as compared with normal prostate epithelial cells, suggesting a role as tumor suppressor for this chaperone (Das et al. 2001). Other contradicting data demonstrated that exogenous FAPB4 bound to fatty acids stimulated invasive behavior of prostate cancer cells in vitro (Uehara et al. 2014).

A negative correlation was observed in bladder cancer patients that showed a decrease in FABP4 transcript with histologic grade and tumor progression (Boiteux et al. 2009). Similarly, it was demonstrated that in hepatocellular carcinoma an increase of FABP4 is accompanied by a decrease of tumor growth and invasiveness (Zhong et al. 2018). This observation seems firm since it was derived from the study of 165 patients, therefore, it is tempting to suggest that positive chaperonotherapy may be a promising experimental approach, for example in animal models, by testing FABP4 as an anti-tumor agent in hepatocellular carcinoma.

#### FABP5

Immunohistochemical analyses of FABP5 performed in normal, benign prostate tumor, and malignant prostatic tumor tissues revealed that over 70% of prostate cancers show increased levels of FABP5 (Adamson et al. 2003), Table 1. This lipid chaperone contributes to both the onset of primary tumor and to metastasization (Morgan et al. 2008). Experimental assays demonstrated that suppression of FABP5 is accompanied by tumor mass reduction and abrogation of metastasization in prostate cancer. These findings also suggest that FABP5 could be considered as a prognostic indicator and a target for therapeutic agents. Similar conclusions were derived from a study of patients with uveal melanoma confirming that FABP5 is associated with progression of some types of tumor and can be considered a prognostic marker (Xu et al. 2020).

A pronounced increase of FABP5 was also observed in breast cancer, and molecular analyses showed a close correlation between the chaperone levels with activation of the epidermal growth factor receptor (EGFR) signalling pathway (Powell et al. 2015). The possible role of FABP5 in carcinogenesis was also proposed for metastatic transformation and stromal-cell interaction in triple-negative breast cancer cells (TNBCs) (Apaya et al. 2020). Negative chaperonotherapy was applied using doxorubicin in combination the phytogalactolipid 1,2-di-O-α-linolenoyl-3-O-β-galactopyranosyl-sn-glycerol (dLGG) to inhibit FABP5 and, thereby, reduce TNBC recurrence.

#### FABP6

An increase of this chaperone occurs in subjects with colon cancer with a profile of FABP6-gene expression closely correlated with the tumor size, location, and invasion depth (Ohmachi et al. 2006), Table 1. A similar feature was observed in renal-cell carcinoma, in which FABP6 mRNA was increased 39-fold in comparison with normal tissue (Schrödter et al. 2016).

#### FABP7

Physiologically, FABP7 is expressed in neural stem cells and radial glia in which it is involved in neurogenesis regulation (Liu et al. 2010), Table 1. In addition, FABP7 has carcinogenic potential because of its capability of promoting cell proliferation. Increased FAPB7 was found in several human cancers such as cystic carcinoma, malignant melanoma, and invasive glioma (Goto et al. 2010; Kagawa et al. 2019). Similar observations were reported for renal carcinoma cells when compared with normal kidney tissue. In this case, an increase of the chaperone levels positively correlated with the aggressiveness of the tumor and with poor survival rate (Nagao et al. 2018). FABP7 overexpression in renal cancer cells triggered a pronounced proliferation rate and activated signal transduction pathways associated to ERK kinase and STAT3 signalling (Nagao et al. 2018). These data suggest that FABP7 can contribute to carcinogenesis and that certain types of renal cancer can be considered chaperonopathies amenable to treatment with anti-chaperone reagents (negative chaperonotherapy).

#### FABP9

A malignant potential has been unveiled for FABP9 in PC3-M prostate cancer cells, in which the chaperone’s mRNA levels are elevated in cultured tumor cells as compared with normal counterparts (Al Fayi et al. 2016), Table 1.

### Neurodegenerative diseases

Neurodegenerative diseases are characterized by irreversible and progressive loss of neuronal cells in specific areas of the brain. Since neurons are cells that normally do not regrow and that cannot be replaced by the body, neurodegenerative disorders are debilitating and non-curable diseases (Dugger and Dickson 2017). Progressive degeneration and death of neurons lead to disturbances in movement (ataxias) or mental functioning (dementia). The etiology and pathogenic mechanism of this heterogeneous group of diseases are still under investigation. It is believed that in the development of neurodegenerative diseases, genetic and environmental factors are involved (Erkkinen et al. 2018). At the cellular level, a series of processes are activated, such as oxidative stress and mitochondrial dysfunction, leading to accumulation of misfolded and aggregated proteins in the brain, with ensuing apoptosis when damage is irreversible (Hetz and Saxena 2017). Due to the high lipid concentration in nervous tissue, another important factor that can lead to pathology in the central nervous system (CNS) is alteration in lipid biosynthesis and transport. Pro-inflammatory signals mediated by, for instance, TNFα and IL-1, promote the formation of atherosclerotic plaques leading to ischemic stroke and altered lipid metabolism with production of reactive oxygen species, all factors that damage the CNS and cause neurological disorders (Adibhatla and Hatcher 2008). Since elevated levels of free fatty acids are cytotoxic, the human body has developed a defense mechanism in the form of small cytoplasmic proteins (FABPs) that bind long chain fatty acids and then ushers them to appropriate intracellular disposal sites. FABP3, FABP5, and FABP7 are specifically localized in neurons and glia (Matsumata et al. 2016). Altered levels of these FABPs are correlated with neurological diseases (Alzheimer’s and Parkinson’s) and other disorders of cognitive function of which the pathophysiological basis is oxidative stress (Choromańska et al. 2011). Generally, through a chain of intracellular signals, the cell senses oxidative stress and reacts by inducing the synthesis of various antioxidant proteins as a defense system, including thioredoxin-1 (Trx-1), a small protein that acts as a major antioxidant in all animal cells.

#### FABP3

This chaperone is involved in synapse formation and in the activity of cholinergic and glutamatergic neurons, and it was found increased in the cerebrospinal fluid of patients with various neurological disorders (Chiasserini et al. 2017), Table 1. Furthermore, FABP3 has been correlated with tau and α-synuclein aggregation, the typical markers of neurodegeneration (Chiasserini et al. 2017). FABP3 is critical for α-synuclein uptake in dopaminergic neurons, preventing the development of synucleinopathies, such as Parkinson’s disease (Kawahata et al. 2019). FABP3 knockout mice (FABP3 KO) showed a reduction in α-synuclein oligomerization and neuronal degeneration. This study highlights the importance of FABP3 in pathogenesis and suggests that FABP3-dependent α-synuclein absorption must be regulated by a mechanism yet to be elucidated. This would be a situation encouraging animal research on the use of FABP3 in positive chaperonotherapy, by administering the chaperone to test its efficacy in controlling the progression of synucleinopathies and similar protein-aggregation pathologies. In addition, FABP3 has potential as biomarker for disease monitoring: epidemiological studies have shown a close association between elevated levels of FABP3 in sera of patients with Parkinson’s and Alzheimer’s disease and disease progression (Chiasserini et al. 2010; Teunissen et al. 2011).

#### FABP5

This chaperone is important for neuron development and appears to have an impact on the levels of decosahexaenoic acid (DHA) in the brain (Pan et al. 2018), Table 1. DHA is an essential fatty acid that has strong antioxidant and neuroprotective capabilities (Pan et al. 2018). The neuroprotective function of this polyunsaturated fat is associated with its potent effect on synaptic transmission and long-term potentiation. Since the brain has a limited ability to synthesize its own DHA, FABP5 mediates the transport of DHA, allowing for proper uptake of the molecule. In this way, there is a connection between an endogenous reduction of DHA levels, caused by a deficiency of FABP5, and a cognitive deterioration (Pan et al. 2018). FABP5 knockout mice (FABP5 KO) show impaired working memory and short-term memory compared to wild-type (WT) mice. This behavioral defect appears to be associated with reduction of endogenous DHA levels in the brain and clearly point to FABP5 as molecular target for investigation, diagnostic, and treatment of pathologies caused by DHA deficiency. In some instances, DHA deficiency could be a chaperonopathy by defect, in which the chaperone FABP5 is either quantitatively or qualitatively deficient, and may benefit by positive chaperonotherapy, consisting in administering the deficient chaperone.

#### FABP7

Although the molecular mechanisms linking FABPs and ROS are poorly understood, studies show that FABP7 knockout mice (FABP7 KO) have high ROS toxicity due to a reduced activation of Trx-1, with a consequent activation of apoptosis signaling molecules, including p38 mitogen-activated protein kinase (MAPK) and increased expression of caspase 3 cleaved (Islam et al. 2019). This suggests that increased levels of FABP7 are in certain diseases a defense mechanism of the cell against stress, Table 1. Probably, FABP7, a strong binder of omega-3 PUFAs, causes an inhibition of the behavioral and brain changes that occur as a consequence of stressful life events (Réus et al. 2018; Sharifi et al. 2011). FABP7 appears to have a protective role against neuronal toxicity and it can be considered as a diagnostic marker for the detection of brain injury from trauma or neurodegenerative diseases (Pelsers and Glatz 2005; Teunissen et al. 2011). These observations encourage investigations on the use of FABP7 in positive chaperonotherapy for controlling certain types of neurological disorders, in which the chaperone is involved but is in concentrations that are not enough to deal with the quantity of stressors present in the cell. In situations like this, administration of FABP7 would merit investigation to test its therapeutic potential in animal models. Similar considerations apply in the following examples.

FABP7 is involved in the absorption and transport of fatty acids, in signal transduction, and in gene transcription and when deficient in astrocytes certain brain functions are altered (Ebrahimi et al. 2016). FABP7 KO mice show not only abnormal dendritic morphology but also a reduced number of excitatory synapses compared to wild-type mice (Ebrahimi et al. 2016). These results suggest that astrocytic FABP7 is important for the growth of the dendritic arbor, the formation of neuronal excitatory synapses, and synaptic transmission, providing new insights that link FABP7-dependent lipid homeostasis and neuropsychiatric disorders (Ebrahimi et al. 2016). Furthermore, astrocyte-FABP7 may function as a modulator of blood-brain barrier (BBB) permeability, after traumatic brain injury (TBI) (Rui et al. 2019). Consequently, the overexpression of FABP7 in conjunction with the upregulation of endothelial Cav-1 may be an endogenous protective response to BBB disruption (Rui et al. 2019).

The data discussed above suggest that FABP3, FABP5, and FABP7 may be used not only as therapeutic targets or agents in chaperonotherapy for patients with disorders, in which the chaperones play an etio-pathogenic role, but also as biomarker of disease. While chaperonotherapy is still at the experimental stage, the use of FABPs as biomarkers in diagnosis, and in the follow-up of patients could become a general practice immediately, for example in patients with brain lesions and degenerative pathologies because these chaperones are easily measurable in blood (Teunissen et al. 2011; Mollenhauer et al. 2007).

## Conclusions and perspectives

Protein chaperones like Hsp60, Hsp70, and Hsp90 and many others, with polypeptides as their known clients (substrates) and interactors can cause diseases, the chaperonopathies, if abnormal in structure, function, location, or quantity. Lipid chaperones, i.e., FABPs whose substrates are lipids, also have the potential for causing diseases if abnormal qualitatively or quantitatively. Here, we propose to include these diseases within a single nosological group, the lipid chaperone chaperonopathies, like that encompassing the chaperonopathies associated with protein chaperones (Macario and Conway de Macario 2005; Macario and Conway de Macario 2007b).

Chaperones assist other molecules, proteins or lipids, to successfully negotiate one or more of these physiological pathways: (a) mature into a functional shape; (b) travel to reach the place of residence either the cytoplasm and membranes or through membranes to enter the various cell compartments including storage spaces; (c) associate with partners to form functional multi-molecular complexes; (d) dissolve pathological aggregates; (e) protect from the denaturing effects of stressors; (f) regain functional status after partial reversible denaturation; and (g) escort irreversibly damaged or unnecessary molecules toward degradation and elimination out of spaces in which they would interfere with physiological biochemical processes. It is, therefore, obvious that chaperones are everywhere inside cells and outside them, and in circulation, and interact with many molecules in all tissues and organs. Consequently, failure of a chaperone is bound to have systemic effects, although with manifestations more marked in the cell tissues in which it is most expressed and needed, as demonstrated by the many chaperonopathies studied that affect predominantly, but not exclusively, the central or peripheral nervous systems, or the muscular system, or the cardio-circulatory apparatus, and so on. Thus, chaperonopathies, whether associated with protein or lipid chaperones, fall under the expertise of a variety of medical specialties with many contact points allowing interaction between a range of scientists and practitioners. This cross-fertilization should facilitate diagnosis, interpretation of clinical and laboratory data, and the implementation of common treatment strategies. Chaperonopathies affecting protein or lipid chaperones are systemic, and considering them within two defined nosological groups leads to a better understanding of their pathogenic mechanisms, what is common to all of them and what is distinctive of each of them as individual entities, or of subgroups with similar properties. This should lead to improvements in patient management and progress in the learning and teaching of these diseases, following a coherent schema on a unified platform.

## Abbreviations

ABCA1: ATP-binding cassette A1
AFABP: Adipocyte fatty acid–binding protein
ATGL: Adipose triglyceride lipase
BBB: Blood-brain barrier
B-FABP: Brain typed FABP
E-FABP: Cutaneous fatty acid–binding protein
CNS: Central nervous system
DHA: Decosahexaenoic acid
dLGG: Phytogalactolipid 1,2-di-O-α-linolenoyl-3-O-β-galactopyranosyl-sn-glycerol
EGFR: Epidermal growth factor receptor
FAs: Fatty acids
FABPs: Fatty acid–binding proteins
FAS: Fatty acid synthase
HCC: Hepatocellular carcinoma
HDL: High-density lipoprotein
H-FABP: Heart fatty acid–binding protein
HNF-1α: Hepatocyte nuclear factor 1 α
HSL: Hormone-sensitive lipase
HSFs: Heat shock factors
Hsps: Heat shock proteins
I-BABP: Ileal bile acid–binding protein
JNK: c-Jun N terminal kinase
LDL: Low-density lipoprotein
MAPK: Mitogen-activated protein kinase
MCP-1: Monocyte chemoattractant protein 1
MDGI: Mammary-derived growth inhibitor
MGL: Monoglyceride lipase
MS: Metabolic syndrome
NF-κB: Nuclear factor κ-light-chain-enhancer of activated B cells
NSAIDs: Non-steroidal anti-inflammatory drug
NSLCLs: Non-small cell lung cancer
PPARγ: Peroxisome proliferator-activated receptor γ
PUFA: Polyunsaturated fatty acid
TBI: Traumatic brain injury
T-FABP: Testis-FABP
TLR4: Toll-like receptor 4
TNBCs: Triple-negative breast cancer cells
TNF-α: Tumor necrosis factor α
Trx-1: Thioredoxin-1
